# Comment on a recent article titled “Circulating level of fatty acid‐binding protein 4 is an independent predictor of metabolic dysfunction‐associated fatty liver disease in middle‐aged and elderly individuals”

**DOI:** 10.1111/jdi.13798

**Published:** 2022-04-13

**Authors:** Mehmet Ilkin Naharci, Ilker Tascı

**Affiliations:** ^1^ Division of Geriatrics Gulhane Faculty of Medicine & Gulhane Training and Research Hospital University of Health Sciences Ankara Turkey; ^2^ Department of Internal Medicine Gulhane Faculty of Medicine & Gulhane Training and Research Hospital University of Health Sciences Ankara Turkey

## Disclosure

The authors declare no conflict of interest.

Approval of the research protocol: N/A.

Informed consent: N/A.

Registry and the registration no. of the study/trial: N/A.

Animal studies: N/A.

To the Editor,

In a recent issue of the *Journal of Diabetes Investigation*, Tanaka *et al*.^1^ reported valuable findings regarding the relationship of metabolic dysfunction‐associated fatty liver disease (MAFLD) and fatty liver index (FLI) with fatty acid binding protein 4 (FABP4) in middle‐aged and older adults. This study expanded the overall understanding of the association of hepatosteatosis with altered cytokine production and the independent predictors of MAFLD.

However, we would like to address some issues that need clarification. We appreciate the efforts of the authors to comprehensively control for some potential confounders. Nevertheless, the physical activity level and renal function that could influence the adjusted analyses were not taken into consideration.

Exercise confers a clear benefit on preventing or delaying the development of chronic diseases. The beneficial effect of exercise occurs in part through changes in the adipose tissue, namely by promoting anti‐inflammatory cytokines and reducing proinflammatory cytokines. A follow‐up study showed that the circulating FABP concentrations decreased after 3 months of exercise therapy with improving body composition and metabolic parameters in obese women^2^.

Another point we would like to discuss is the relationship of serum FABP4 level with renal function. A prospective study of participants with type 2 diabetes mellitus showed that an elevated serum FABP level was a predictor for adverse renal outcomes over a median follow‐up of 5 years^3^. The serum FABP4 level was significantly elevated in parallel with a reduced estimated glomerular filtration rate in patients with chronic kidney disease^4^. The FABP4 levels were also shown to be highly dependent on kidney function in an experimental model^4^. In addition, elevated urinary FABP4 was significantly associated with the progression of renal dysfunction^4^. As noted in the current article, the mean age of the participants was 65 ± 15 years, suggesting that the study consisted of a significant number of older subjects. The prevalence of chronic kidney disease is significantly increased in older adults with advancing age and may be encountered earlier in subjects with MAFLD.

In conclusion, the authors have demonstrated a relationship between MAFLD and serum FABP4 level through rigorous research. However, the conclusions would be wiser if the authors could provide further adjusted data on physical activity and renal function. Consideration of confounders that have clinical impacts may also be helpful in the development of novel therapeutic actions.
